# Supplementary figures and images for: The small tumor antigen of Merkel cell polyomavirus accomplishes cellular transformation by uniquely localizing to the nucleus despite the absence of a known nuclear localization signal
Source: Virol J. 2024 Jun 3;21:125. doi: 10.1186/s12985-024-02395-x (PMC11149282; doi:10.1186/s12985-024-02395-x)

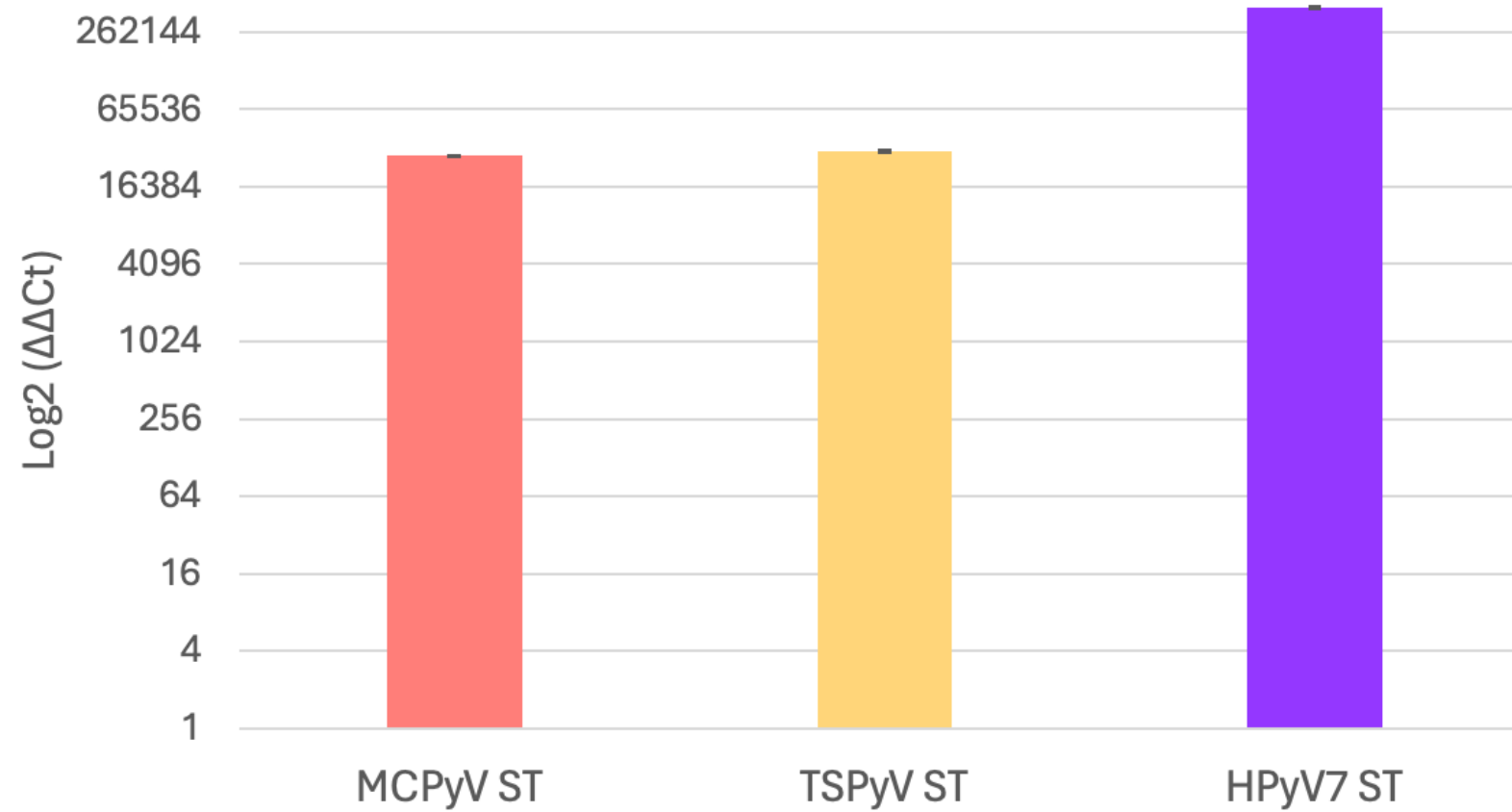

Supplement: Supplementary file 1 — Supplementary Material 1. [file 12985_2024_2395_MOESM1_ESM.pdf]
