# Supplementary material for: The small tumor antigen of Merkel cell polyomavirus accomplishes cellular transformation by uniquely localizing to the nucleus despite the absence of a known nuclear localization signal
Source: Virol J. 2024 Jun 3;21:125. doi: 10.1186/s12985-024-02395-x (PMC11149282; doi:10.1186/s12985-024-02395-x)
Supplement: Supplementary file 2 — Supplementary Material 2. [file 12985_2024_2395_MOESM2_ESM.pdf]

```
HPyV7_ST        MDKLLGRDEVKELMELIGLNMACWGNLPLIQHKVRLASKKYHPDKGGDPQKMQRLNVLKD      60
MCPyV_ST        MDLVLNRKEREALCKLLEIAPNCYGNIPLMKAAFKRSCLKHHPDKGGNPVIMMELNTLWS      60
TSPyV_ST        MDKFLSREESLELMDLLQIPRHCYGNFALMKINHKKMSLKYHPDKGGDPEKMSRLNQLWQ      60
                ** .*.*.*   * .*: :   *:**: *::   :  . *:******:*  * .** * .

HPyV7_ST        KLEATLRDQRSGSPMWHYSSDEVSFWDIE-LTVGEFLGP-EFNRKKVWNYNLCVVQGL--      116
MCPyV_ST        KFQQNIHKLRSDFSMF-DEVSTKFPWEEY-GTLKDYMQS-GYNA-RFCRGPGCMLKQLRD      116
TSPyV_ST        KLQEGIYNARQEFPTSFSSQVGSWYWEANLISLKEYFGKKKYDENVIKHWPQCAEKAL--      118
                *::  : . *.       .      *:    :: :::    ::   . .   *  : *

HPyV7_ST        RACCCIHCILKRKHKKKAKEYAKDQRGPLSWGKCWCFHCYLNWFGVERSEES-FMWWSHI      175
MCPyV_ST        SKCACISCKLSRQHCSLK---TLKQKNCLTWGECFCYQCFILWFGFPPTWES-FDWWQKT      172
TSPyV_ST        KECKCLTCKIGLQHFVYK---QMHQKKCVVWGECFCYKCYCAWFGEDLYCLDSLWAWSCI      175
                  * *: * :  :*         .*:  : **:*:*::*:  ***      . :  *.

HPyV7_ST        IFQTPMDVLNLWGQLNLL-----   193
MCPyV_ST        LEETDYCLLHLHLF---------   186
TSPyV_ST        VGEVDFHLVNLYLRVNQGFNWGK   198
                : :.   :::*
```
